# Effectiveness of Diabetes Self-Management Education on Quality of Life in Diabetic Elderly Females

**DOI:** 10.5539/gjhs.v7n1p10

**Published:** 2014-07-29

**Authors:** Marzieh Kargar Jahromi, Somayeh Ramezanli, Leila Taheri

**Affiliations:** 1 Community Health Nursing, faculty member of Jahrom University of Medical Sciences, Jahrom, Iran; 2 Medical-Surgical Nursing, Jahrom University of Medical Sciences, Jahrom, Iran; 3 Pediatric Nursing, Jahrom University of Medical Sciences, Jahrom, Iran

**Keywords:** diabetes, elderly females, Quality of Life, self-management education

## Abstract

**Introduction:**

Diabetes is a chronic illness that requires continuing medical care and patient self-management education to reduce the risk of long-term and acute complications. The aim of the present study was to determine the effectiveness of diabetes self-management education on quality of life in elderly females with diabetic mellitus (type 2) in Shiraz, Iran, 2013.

**Method:**

The study was conducted from January to April 2014 at the Jahandidegan center, a day center affiliated to Shiraz welfare organization. The instrument used for the study was the Quality of life Questionnaire (WHO QOL-BREF) SF26. After an explanation of the aim of the study by the researcher, 90 participants with all the required criteria and G.H.Q score ≤ 23 were selected as the study sample for the intervention. Participants divided into experimental and control groups, and completed WHO QOL-BREF before the intervention, 2 and 3 months after the last session of education.

**Result:**

It is shown that 2 and 3 months after the intervention, QOL scores had a significant difference between the two groups. In other words, the training sessions improved the score of QOL in the intervention group (P < 0.001) versus control group (P = 0.5).

**Conclusion:**

The Behavioral Intervention Program significantly improved the quality of life outcomes of the diabetic elderly females. Thus, it is concluded that the diabetic individuals can be significantly improved following instruction by health care providers.

## 1. Introduction

Over the past 20 years, there has been an explosive increase in the number of cases of diabetes (Khan et al., 2011). The International Diabetes Federation (IDF, 2011) has recently estimated that in 2030, 9.9% of the adult population worldwide will have diabetes (552 million).

Type 2 diabetes accounts for at least 90% of diabetes cases in developed countries (Huse, Oster, & Killen, 1989). Iran has a 7.7% (approximately 2 million adults) population rate of prevalence of diabetes within the age range, from 25 to 64 (Esteghamati et al., 2009). This rather high prevalence is seemingly rising (Rathmann, & Giani, 2004). This increase is likely to have a significant public health impact given the high rates of acute myocardial infarction, heart failure, stroke, and death that follow diabetes (Khan et al., 2011), and implies a substantial burden on both the individual and the healthcare system (Gody, 2002). Diabetes is also associated with significant health care costs (National Diabetes Fact Sheet, 2007). The aggregate annual direct costs of diabetes in Iran, is estimated to be 590.676 ± 65.985 million US dollars (Esteghamati et al., 2009).

The adverse effects of diabetes on health-related quality of life (HRQOL) are described in a growing body of literature (Dasbach, R. Klein, B. E. Klein, & Moss, 1997). The impact of diabetes complications adversely affects self-care behaviors, glycemic control and quality of life (Grigsby, Anderson, Freed land, Clouse, & Lust man, 2002; Clouse et al., 2003). The greater risk of complications in women compared to men may be due to differences in how women experience and manage their diabetes (Ellis et al., 2004).

Because of the considerable societal burden of type 2 diabetes, its impact on HRQOL is a public health issue of concern to patients, families, employers, health care providers, and payers (Mokdad, Ford, & Bowman, 2000). Yet little research has been conducted on diabetes in elderly populations. Because diabetes is a silent disease, with no sudden or exact date of onset, it is difficult to design studies of disease incidence. Lastly, mortality is known to be higher in people with diabetes than in those without it, and diabetes accounts for considerable morbidity and mortality in the elderly population (Jansson, Andersson, & Svardsudd, 2009.

There is strong evidence to support the efficacy and cost effectiveness of programs that help improve glycemic control and reduce complications in patients with type 2 diabetes mellitus (T2DM) (Stratton et al., 2000).

Some literatures suggest that health promoting behaviors of older adults offer the potential for improving their health status and HRQOL. In which one of the most important duties of health professionals is health education and propagation of healthy behaviors. Studies show that health education has a significant impact on awareness of people (Delshad & Bahri, 2004). Although increasing age is a risk factor for the development of diabetes and its vascular complications, few studies have focused specifically on the impact of education about diabetes care in elderly populations (Gregg et al., 2012).

For patients with chronic disease, there is growing interest in “self-management” programs that emphasize the patients’ central role in managing their illness (Kate et al., 2001). While it is well established that diabetes self-management education (DSME), a complex health intervention, is generally effective at enhancing self-care behaviors (Gary et al., 2003), improving glycemic control (Pimouguet, Le, Thiebaut, Dartigues, & Helmer, 2011), lowering health care costs (Li, P. Zhang, Barker, Chowdhury, & X. Zhang, 2010), and improving quality of life (Gary et al., 2003). The specific impact of DSME features on outcomes has not been thoroughly evaluated (Blue & Black, 2005), particularly for specific cultural and gendered populations. For instance, research shows that women have different self-management education needs compared with men. These findings suggest that men and women with diabetes may have different DSME needs and that different cultures may respond better to various DSME intervention features than others. DSME programs are complex interventions with various content and delivery components necessary for the education and skills building required for diabetes self-management. However, limited efforts have been made to investigate which intervention features are associated with a positive outcome (Brown et al., 2000). Based the above, this study aimed to evaluate the effectiveness of diabetes self-management education on quality of life in elderly females with diabetic mellitus (type 2) in Iran.

## 2. Methods and Materials

### 2.1 Study Design

This study was an interventional study with simple random sampling to evaluate the effectiveness of diabetes self-management education on quality of life in elderly females with type 2 diabetic mellitus in Iran.

### 2.2 Study Setting and Sample

The study was conducted from January to April 2014 at the Jahandidegan center, a day center affiliated to Shiraz welfare organization. The center has about 5000 registered members aged above 60. The members participated voluntarily in various activities in the center. The inclusion criteria were diabetic female adults aged 60–74 years old, who were members of the center and who get the score of 23 or less in General Health Questionnaire. The exclusion criteria were: having a personal crisis during the intervention, or suffering from severe physical or psychological disorders, participating in other activities during the intervention (e.g., sports/physical activities or counseling). On the basis of these criteria 90 older females were selected randomly to participate in the study.

### 2.3 Data Collection

The instrument used for the study was the Quality of life Questionnaire (WHO QOL-BREF) SF26. After an explanation of the aim of the study by the researcher, 90 participants with all the required criteria and G.H.Q score ≤ 23 were selected as the study sample for the intervention. Participants divided into experimental and control groups, and completed WHO QOL-BREF before the intervention, 2 and 3 months after the last session of education. The verbal and written informed consent was taken from the participants before the intervention.

### 2.4 The Questionnaire

The WHOQOL-BREF is a 26-item instrument consisting of four domains: physical health (7 items), psychological health (6 items), social relationships (3 items), and environmental health (8 items); and two overall QOL and general health items. The physical health domain includes items on mobility, daily activities, functional capacity and energy, pain, and sleep. The psychological domain measures self-image, negative thoughts, positive attitudes, self-esteem, mentality, learning ability, memory and concentration, religion, and the mental status. The social relationships domain contains questions on personal relationships, social support, and sex life. The environmental health domain covers issues related to financial resources, safety, health and social services, living physical environment, opportunities to acquire new skills and knowledge, recreation, general environment (noise, air pollution, etc.), and transportation. The instrument used for the study was the Quality of life Questionnaire (WHO QOL-BREF) SF26. The validity and reliability of it was performed in many studies, e.g., have validated a Farsi version on a community dwelling sample. The questionnaire alpha has reported as 0.89 which concludes that the Farsi version was a reliable and valid instrument for QOL in Iran (Nedjat, Montazeri, Holakouie, Mohammad, & Majdzadeh, 2008).

### 2.5 Intervention

The experimental group was divided into 3 sub-groups (15 in each) and 8 group interaction sessions were held weekly for each sub-group. For this study 8 topics were selected for discussion. The topics were: nutrition, stress management, physical activity, sleep and rest, safety, glycemic control, and improving self-care (all items were in older segment). At the end of the sessions, one of the participants voluntarily summarized the discussed materials and the members were notified about the topic for the next session.

At first the coordinator designed the chairs in semi-circle forms so that the participants can see and interact with each other. Then the topic was announced and the participants were asked to explain about their experiences on the topic. The coordinator managed the time and controlled the group participation.

### 2.6 Data Analysis

The results were analyzed by SPSS version 16. The data were examined using Paired t-test and repeated measurement.

## 3. Result

Overall, 73.7% of the participants were married, 70.4% educated under diploma, 72.2% had given birth to four children or more and had diabetic mellitus for at least 5 years ago. The mean age and body mass index of the subjects are presented in Table 1. As shown in Table 2, the mean score for QOL before the intervention in the two groups is almost the same (P = 0.4). The results after the intervention are shown in Table 3. It is shown that 2 and 3 months after the intervention, QOL score has a significant difference between the two groups (P = 0.012). In other words, the training sessions improved the score of QOL in the experimental group (P < 0.001) versus control group (P = 0.5).

**Table 1:** Mean and standard deviation of age and body mass index in the two groups

Group	Experimental		Control		P-value

Variable	M	SD	M	SD	
**Age**	**66.76**	**5.35**	**67.71**	**4.12**	P=0.5
**BMI**	**25.07**	**2.85**	**23.85**	**3.71**	P=0.2

**Table 2:** Comparison of mean of QOL score before the intervention in the two groups

Time	Before the intervention		P-value

Group	M	SD	
**Experimental**	41.65	19.41	P = 0.4
**Control**	39.32	18.43	

**Table 3:** Comparison of mean of QOL score after the intervention in the two groups

Time	2m later		3m later		P-value

variable Group	M	SD	M	SD

**Experimental**	**54.50**	**20.35**	**59.75**	**19.34**	P=0.012
**Control**	**38.43**	**18.40**	**37.90**	**18.03**		

## 4. Discussion

Diabetes is a chronic illness that requires continuing medical care and patient self-management education to reduce the risk of long-term and acute complications (American Diabetes Association, 2009). More than 171 million people have diabetes in the world and this number is expected to reach up to 366 million in 2030 (Wild, Roglic, Green, Sicree, & King, 2004). As prevalence of diabetes in elderly people has increased and their life expectancy has shortened (Boyle et al., 2001)

Patients with elevated blood glucose have an increased risk of numerous microvascular and macrovascular complications, all of which may negatively affect HRQOL. Results from this study indicate that even mild complications in patients with type 2 diabetes can have a profound effect on the patients’ perceived HRQOL as measured using the well-validated and widely used SF-36 questionnaire (Lioyd, Sawyer, & Hopkinson, 2001).

Basically the remedy of diabetes, in a large extent, depends on patients’ self-care. Patients are expected to control 95% of diabetes themselves, and make a significant change in their life style. They should change simultaneously their diet, physical exercise, and individual control of their blood sugar (Clarke, 2002). However recent studies, using national representative samples have shown that few patients follow multiple self-care behaviors at recommended levels (Nwasuruba, Khan, & Egede, 2007; Nwasuruba, Osuagwu, Bae, Singh, & Egede, 2009).

Leaders in diabetes education emphasize the importance of using health behaviors change theories and models to drive diabetes self-care efforts, because they are more likely to effectively change behavior and maintain behavior change (Osborn & Fisher, 2008).

In our study, the effects of 8 training classes, showed a considerable increase in QOL score, 2 and 3 months after intervention, for the subjects attending in the training classes.

Our results support the Gavgani study that revealed, the total self-care, and the self-care of diet and exercise has been increased significantly in the experimental group. Behavior change was qualified by significantly decreased HbA1C in the experimental group, and the weight loss either was not significant, the high effect size in the experimental group revealed the impact of intervention on decreasing the weight (Gavgania, Poursharifib, & Aliasgarzadehcd, 2010).

Forouhari et al. (2010) did a study entitled “the Effects of training on quality of life in 62 menopause females”. The results showed a significant increase in the mean scores in all quality of life dimensions (Vasomotor, psychological-social, physical, and sexual) in intervention group.

Our results support the Oh et al. (2005) and Cardozo (2004) studies that showed a significant improvement in the quality of life outcomes in elderly people. The results of all studies showed a significant improvement on the quality of life outcomes.

The result of kate’ s study showed at 1 year, participants in the program experienced statistically significant improvements in health behaviors (exercise, cognitive symptom management, and communication with physicians), self-efficacy, and health status (fatigue, shortness of breath, pain, role function, depression, and health distress) and had fewer visits to the emergency department (Kate et al., 2001).

Health care providers need to be sensitive to these issues and identify better ways to evaluate and discuses DM with their patients. In addition, they can play an important role in teaching patients about their health condition, treatment options, and disease management.

## 5. Conclusion

The 8 week training classes significantly improved the quality of life outcomes of the elderly females with diabetic mellitus (type 2). Thus, it is concluded that the individuals with diabetic mellitus can be significantly improved following teaching from health care provider.
